# High-Risk Clinicopathological and Genetic Features and Outcomes in Patients Receiving Neoadjuvant Radiochemotherapy for Locally Advanced Rectal Cancer

**DOI:** 10.3390/cancers13133166

**Published:** 2021-06-24

**Authors:** Sofía del Carmen, Luís Antonio Corchete, Cristina González Velasco, Julia Sanz, José Antonio Alcazar, Jacinto García, Ana Isabel Rodríguez, Rosario Vidal Tocino, Alba Rodriguez, Luis Alberto Pérez-Romasanta, José María Sayagués, Mar Abad

**Affiliations:** 1 Department of Pathology and IBSAL, University Hospital of Salamanca, University of Salamanca, 37007 Salamanca, Spain; scarmen@saludcastillayleon.es (S.d.C.); cgvelasco@saludcastillayleon.es (C.G.V.); jsrepetto@saludcastillayleon.es (J.S.); arodriguezcarr@saludcastillayleon.es (A.R.); 2 Cancer Research Center and Hematology Service and IBSAL, University Hospital of Salamanca, University of Salamanca, 37007 Salamanca, Spain; lacorsan@usal.es; 3 General and Gastrointestinal Surgery Service and IBSAL, University Hospital of Salamanca, University of Salamanca, 37007 Salamanca, Spain; jaalcazar@yahoo.es (J.A.A.); jgarcia@usal.es (J.G.); 4 Radiation Oncology Service and IBSAL, University Hospital of Salamanca, University of Salamanca, 37007 Salamanca, Spain; airodriguezg@saludcastillayleon.es (A.I.R.); lapromasanta@saludcastillayleon.es (L.A.P.-R.); 5 Medical Oncology Service and IBSAL, University Hospital of Salamanca, University of Salamanca, 37007 Salamanca, Spain; mrvidal@saludcastillayleon.es

**Keywords:** locally advanced rectal cancer, neoadjuvant radiochemotherapy, TNM, SNP arrays

## Abstract

**Simple Summary:**

The overall genomic copy number changes profile of three subgroups of locally advanced rectal carcinoma patients with significantly different response to neoadjuvant treatment with radiochemotherapy (ranging from complete to poor- or no-response) was analyzed and compared with a set of normal samples from healthy individuals with negative colonoscopies from the Castilla y León (Spain) region. We identified and validated a novel genetic signature, which combined with clinicopathological features, predicts response to neoadjuvant treatment and clinical outcome.

**Abstract:**

Administering preoperative radiochemotherapy (RCT) in stage II-III tumors to locally advanced rectal carcinoma patients has proved to be effective in a high percentage of cases. Despite this, 20–30% of patients show no response or even disease progression. At present, preoperative response is assessed by a combination of imaging and tumor regression on histopathology, but recent studies suggest that various genetic abnormalities may be associated with the sensitivity or resistance of rectal cancer tumor cells to neoadjuvant therapy. In the present study we investigated the relationship between genetic lesions detected by high-density single-nucleotide polymorphisms (SNP) arrays 6.0 and response to neoadjuvant RCT, evaluated according to Dworak criteria in 39 rectal cancer tumors before treatment. The highest frequency of copy-number (CN) losses detected corresponded to chromosomes 18q (*n* = 27; 69%), 1p (*n* = 22; 56%), 15q (*n* = 19; 49%), 8p (*n* = 18; 48%), 4q (*n* = 17; 46%), and 22q (*n* = 17; 46%); in turn, CN gains more frequently involved chromosomes 20p (*n* = 22; 56%), 8p (*n* = 20; 51%), and 15q (*n* = 16; 41%). There was a significant association between alterations in the 1p, 3q, 7q, 12p, 17q, 20p, and 22q chromosomal regions and the degree of response to therapy prior to surgery. However, 4q, 15q11.1, and 15q14 chromosomal region alterations were identified as important by five prediction algorithms, i.e., those with the greatest influence on predicting the tumor response to treatment with preoperative RCT. Multivariate analysis of prognostic factors showed that gains on 15q11.1 and carcinoembryonic antigen (CEA) levels serum at diagnosis were the only independent variables predicting disease-free survival (DFS). Lymph node involvement also showed a prognostic impact on overall survival (OS) in the multivariate analysis. A deep-learning-based algorithm showed a 100% success rate in predicting both DFS and OS at 60 months after diagnosis of the disease. In summary, our results indicate the existence of an association between tumor genetic abnormalities at diagnosis, response to neoadjuvant therapy, and survival of patients with locally advanced rectal cancer. In addition to the clinical and biological characteristics of locally advanced rectal cancer patients, these could be used in the future as therapeutic and prognostic biomarkers, to identify patients sensitive or resistant to preoperative treatment, helping guide therapeutic decision-making. Additional prospective studies in larger series of patients are required to confirm the clinical utility of the newly identified biomarkers.

## 1. Introduction

Surgery is currently the key stage in the treatment of locally advanced rectal cancer (LARC), although there is growing evidence from randomized clinical trials that administering preoperative radiotherapy combined with chemotherapy in stage II-III tumors produces a significant reduction in tumor size, tumor stage, and local recurrence rates [1], increasing the rate of sphincter-conserving surgery, survival and, consequently, improving the quality of life of patients with LARC. However, the disease exhibits a spectrum of response to radiochemotherapy (RCT), ranging from complete to poor or no response. According to the various published series, it is expected that 5–25% of patients achieve complete remission (complete absence of tumor cells) and that 40–60% will achieve a significant decrease in tumor mass. Conversely, it is estimated that between 20 and 30% of patients do not respond to treatment [2] and tumor progression is found in a minority of cases [3]. It is not known which types of tumor are more radiosensitive and what factors determine a better response to preoperative RCT. At present, staging with imaging techniques is a well-accepted approach, and the one most commonly used to evaluate response to RCT prior to surgery. Rectal magnetic resonance imaging (MRI) plays a key role in the pre- and post-treatment evaluation of rectal cancer, assisting the multidisciplinary team in tailoring the most appropriate treatment option [4]. In this sense, several MRI biomarkers have been proposed for identification of complete responders. In particular, automatic fibrosis quantification with MRI for its high accuracy should be noted [5]. The study of postoperative blood samples for tumor-specific DNA molecules (ctDNA) has also showed clinical utility for both predict pathological responses to preoperative therapy and detect minimal residual disease after surgery, being a promise of a novel approach to evaluate recurrence risk in patients with LARC. Murahashi et al. [6] and others [7] showed that preoperative ctDNA levels are significantly consistent with the degree of response to neoadjuvant treatment, showing that ctDNA can accurately reflect the real-time tumor burden. Recently, Tie et al. [8] reported that postoperative ctDNA analysis stratifies patients with LARC into subsets that are either at very high or at low risk of recurrence, independent of conventional clinicopathological risk factors.

Although there is evidence of the possible benefit of preoperative RCT, the response to treatment is demonstrably variable, and at present there are no sensitive methods or predictive factors to evaluate this response. For this reason, rectal cancer treatment involves surgery, regardless of the outcome of neoadjuvant treatment. However, it is reasonable to believe that performing radical/aggressive surgeries in patients who achieve complete remission could be avoided, as some studies have suggested. Habr-Gama et al. [9] compared the evolution of patients who underwent surgery, and for whom analysis of the resected tissue showed complete remission, with a series of patients with complete clinical remission (defined as the total disappearance of the confirmed tumor, assessed by endoscopy) who were not operated on, but found no significant differences in the rate of recurrence or in the frequency of distant metastasis 10 years after treatment. The availability of more sensitive methods for evaluating the response to RCT treatment together with the identification of the subgroup of tumors resistant to RCT will avoid the need to subject these patients to a treatment that is not without risk of morbidity and that does not benefit them, nor save the health service time and resources.

The predictive value of molecular markers for response to treatment with RCT in LARC is under debate, and numerous associations of several genetic abnormalities with tumor sensitivity to RCT have been proposed [5,6,7,8,9]. Among the most controversial results reported in the literature are those concerning the most informative predictors of response to neoadjuvant therapy [10]. The variability in response to RCT could be due, in part, to the substantial intratumoral heterogeneity present in LARC, where different clones coexist at variable frequencies in a tumor sample, only some of which are potentially involved in tumor sensitivity or resistance to RCT administered prior to surgery [11]. We [12] and others [11] have identified important differences among cases showing a different grade of response to neoadjuvant therapy with respect to the patterns of intratumoral clonal evolution detected by fluorescence in situ hybridization (FISH), particularly the cytogenetic profiles of the ancestral tumor cell clones for chromosomes 1, 11p, 12p, and 17p. The limited resolution of the molecular techniques used is another important determinant. Chen Z et al. [13] found a greater frequency of losses of 12p13.31 when assessed by comparative genomic hybridization (CGH arrays) in 25 (26%) responder patients of the 95 cases studied. Molinari et al. [14] identified several chromosomal regions by using CGH arrays associated with the preoperative RCT response. More recently, the availability of high-density single-nucleotide polymorphism (SNP) arrays has facilitated the identification of small regions of chromosomal gains and losses because of its higher resolution (down to 2.5 kb), and provides new opportunities for identifying novel cancer genes involved in tumor sensitivity or resistance to RCT administered prior to surgery in patients with LARC.

In the present study we used SNP arrays 6.0 with a median distance between interrogated SNPs of 680 bases to map genetic lesions present at diagnosis in 39 LARC tissue biopsies. Our primary goal was to identify the commonly gained and/or deleted genes in the altered chromosomal regions and to investigate their potential association with response versus resistance to RCT administered prior to surgery, as assessed by the Dworak regression system [15]. To evaluate the reproducibility of the SNP array results, we performed parallel interphase FISH analyses of the same tumor samples using five probes directed against the most frequently altered chromosomal regions [12].

## 2. Materials and Methods

### 2.1. Patients and Samples

Thirty-nine patients (28 men and 11 women; median age of 69 years, range 39–88 years) diagnosed with locally advanced rectal cancer at the University Hospital of Salamanca (Salamanca, Spain) between May 2006 and April 2014 were included in this study. Before treatment was given, patients were grouped according to the *u*TNM classification using imaging techniques, for example, rigid rectoscopy endorectal ultrasound, colonoscopy, computed tomography (CT), and magnetic resonance imaging (MRI). The absence of metastatic disease was a requisite for recruitment. The most relevant clinical and laboratory characteristics of the patients are summarized in Table 1 and described in more detail in Supplementary Table S1, including the adjuvant treatment administered to each of the patients. In every case, radiochemotherapy consisting of long-course radiotherapy with 50.4 Gy administered in 25–28 fractions, plus capecitabine (800–825 mg/m^2^), were given prior to surgical removal of the tumor. On the latter occasion, the degree of response was scored from grade 0 (absence of tumor regression) to grade 4 (complete tumor regression), following the Dworak system (Table 1).

Overall, 39 pretreatment tissue biopsy samples were analyzed by SNP arrays. All samples were sequentially fixed, stained with hematoxylin and eosin, and microscopically evaluated to confirm the presence of tumor cells (≥65% epithelial tumor cells) and to assess the quality of the samples to be used for SNP array analyses. Tumor DNA was extracted from representative areas of freshly frozen tumor tissues for the SNP array studies. DNA was extracted using a QIAamp DNA mini kit (Qiagen, Hilden, Germany), following the manufacturer’s instructions. Laboratory analyses were performed blinded to clinical outcomes in order to ensure the impartiality of results.

The study was approved by the Local Ethics Committee of the University Hospital of Salamanca (PI23/03/2018; Salamanca, Spain) on 9 March 2018 and informed consent was given by each individual before entering the study. All procedures involving human participants were performed in accordance with the ethical standards of the institutional and/or national research committee and with the 1964 Helsinki declaration and its later amendments or comparable ethical standards.

### 2.2. SNP Array Studies

Each DNA sample obtained from tissue biopsies of primary tumors was hybridized to the Genome Wide Human SNP Array 6.0 (Affymetrix, Santa Clara, CA, USA); for this purpose, 500 ng of DNA per array were used, in accordance with the manufacturer’s instructions. Fluorescence signals were detected using the Affymetrix GeneChip Scanner 3000 (Affymetrix), and average genotyping call rates of 96.73% (range, 92.32–99.44%).

Log_2_ copy number (CN) ratio values were extracted from the raw CEL files using the Genotyping Console (v.4.2.0.26) and Chromosome Analysis Suite (ChAS) software (v.4.2.0.80) supplied by Thermo Fisher Scientific, Inc. (Waltham, MA, USA), using 160 healthy tissue samples from individuals of the Castilla y León (Spain) region, kindly provided by the Spanish National DNA Bank Carlos III (Salamanca, Spain), as a diploid reference. CN outliers were reduced by winsorization and the resulting values were segmented using an appropriate gamma value of 40 by the piecewise constant fragments algorithm (PCF). These two processing steps were performed using the copynumber package (v.1.26.0) (23442169) in R (v.3.6.3). Minimal common regions (MCRs) of gain and loss were identified using GISTIC (v.2.0.23) (21527027). Gained and lost segments were defined as regions with a mean log_2_ CN ratio ≥0.1 or ≤−0.1, respectively, and at least 25 markers, with a maximum length of 0.5 times the corresponding chromosomal arm. All the MCRs reported in this work were statistically significant, with values of *q* < 0.05.

### 2.3. Survival Analysis

Overall survival (OS) and disease-free survival (DFS) curves were plotted according to the Kaplan–Meier method, and the Mantel–Cox (log-rank) test was used to establish the statistical significance of the differences between survival curves. Multivariate Cox regression models of the prognostic factors of OS and DFS were developed from initial models including solely the variables significantly associated with OS or DFS in the corresponding univariate analyses. We calculated the variance inflation factor (VIF) to estimate multicollinearity between all the variables studied using the car package (v.3.0-11) [16] in R. Highly collinear variables were excluded from further analysis. The proportionality of risk was calculated for each variable in the Cox model using the Schoenfeld test available in the survminer package (v.0.4.9) [17].Univariate and multivariate survival analyses were performed in R using the survival package (v.3.2-7) [18]. The predictive value of the selected variables was established at 12, 36, and 60 months. Training and validation sets were established by random assignation of two-thirds and one-third of the samples, respectively, using the rannum permutation simulation tool available in SIMFIT (v.7.5.4, https://www.simfit.org.uk/, accessed on 21 June 2021). Survival probability at the selected times was predicted using the pec R package (v.2020.11.17) [19]. The best prediction was determined based on the values of accuracy, precision, sensitivity, and specificity.

### 2.4. Prediction of Response to Preoperative Radiochemotherapy (RCT)

Dworak response prediction was performed with the training and test sets created in the survival prediction analysis, initially using the variables selected in the Cox regression models. We also selected variables by measuring the contribution of the MCRs and clinical variables in the Dworak response using five methods implemented in R: Boruta (v.7.0.0) [20], xgboost (v.1.3.2.1) [21], relative importance (from the relaimpo package [v. 2.2–3] [22], DALEX (v.2.1.1) [23], and vita (v.1.0.0) [24]. Once the importance of the variables had been determined, we analyzed the response prediction by considering either the initial batch of variables or the top three variables according to their contribution to the response. Five prediction algorithms were used for this purpose: (1) weighted Support Vector Machines (wSVM) and (2) unweighted Support Vector Machines (SVM) from the e1071 package (v.1.7–4) [25], (3) Partial Least Squares (PLS) in SIMFIT [26], (4) K-Nearest Neighbors (KNN), and (5) Random Forest (RF) algorithms from the caret package (v.6.0–86) [27]. In the case of the wSVM and SVM methods, the optimal kernel was established using the OptimClassifier package (v.0.1.5) [28] while the optimal values for the cost and gamma parameters were calculated using the tune function available in the e1071 package. The best prediction model was determined on the basis of the success rates, overall, and by response group.

### 2.5. Interphase Fluorescence In Situ Hybridization (FISH) Studies

In all cases, FISH studies were performed on an aliquot of the single-cell suspension prepared from the tumor sample. A set of five locus-specific FISH probes directed against DNA sequences localized in four human chromosomes (Vysis Inc, Downers Grove, IL, USA), specific to the chromosomal regions that most frequently feature gains or deletions in sporadic colorectal cancer [26,27], were systematically used to validate the results obtained with the SNP arrays (Supplementary Table S2). The methods and procedures used for the FISH studies have been described in detail previously [29,30].

### 2.6. Other Statistical Methods

Continuous variables were summarized as the mean, standard deviation (SD) and range; dichotomous variables were summarized as frequencies. The statistical significance of group differences was assessed by Student’s *t* and Mann–Whitney *U* tests for continuous variables, depending on whether they were normally or non-normally distributed, respectively. For qualitative variables, the χ^2^ test or the Fisher exact test were applied (cross-tab; SPSS), when appropriate. Statistical significance was considered to be present once *p* values (or, where appropriate, Pearson-corrected *p* values) were <0.05 (IBM, Inc., Armonk, NY, USA).

## 3. Results

### 3.1. Clinical and Biological Characteristics of Locally Advanced Rectal Cancer (LARC) before and after Preoperative Radiochemotherapy (RCT)

We found statistically significant differences in several of the clinical and pathological characteristics of the 39 patients studied before and after preoperative RCT (Table 1). Thirty-four patients (87%) showed some sign of tumor regression, and 5 (13%) showed no regression according to Dvorak grade. 67% and 31% of the patients were diagnosed as stages T3 and T4 pre-treatment, respectively; only 43% were T3 and none were T4 after surgery (*p* < 0.001). In addition, 31 patients (80%) were suspected of having metastatic lymph nodes prior to neoadjuvant treatment, and only 12 had positive lymph nodes (31%) in their surgical specimens (*p* < 0.001). The TNM stage was significantly more likely to be lower after treatment (*p* < 0.001), given that the majority of tumors (82%) were stage III before treatment, while this proportion dropped to 28% after RCT. Likewise, carcinoembryonic antigen (CEA) serum levels of patients studied were significantly likely to be lower after neoadjuvant treatment of the disease (10 patients [41%] pre-treatment vs. 5 patients [13%] after tumor surgery with CEA serum levels ≥5 ng/mL; *p* = 0.005). No significant differences were found for any of the other characteristics analyzed.

### 3.2. Distribution of Chromosomal Alterations in LARC before Preoperative RCT

**Figure 1:**
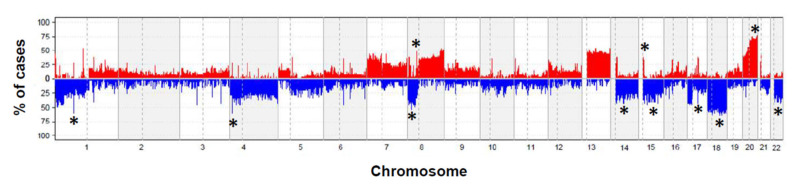
Locally advanced rectal carcinoma genome for the 39 patients genotyped on the Affymetrix SNP array 6.0 platform. A summary plot showing the frequency of gains (in red below zero values on the *x*-axis) and losses (in blue above zero values on the *x*-axis) identified in samples obtained prior to therapy are shown for the whole genome. Those chromosomal regions most commonly showing recurrent losses and gains were localized in chromosomes 1p, 4q, 8p, 14q, 15q, 17q, 18q, and 22q, and in the 8q, 15q, and 20q chromosomal regions, respectively (*q* < 0.001) and are indicated *.

Overall copy number (CN) changes for at least one chromosomal region were detected in the tumors studied (Figure 1). The highest frequencies of CN losses were detected in chromosomes 18q (*n* = 2; 72%), 1p (*n* = 20; 56%), 8p (*n* = 18; 50%), 15q (*n* = 17; 47%), 17q (*n* = 17; 17%), 22q (*n* = 17; 47%), 14q (*n* = 16; 44%), and 4q (*n* = 16; 44%); in turn, CN gains more frequently involved chromosomes 20p (*n* = 21; 58%), 8p (*n* = 17; 47%) and 15q (*n* = 15; 42%) (Table 2). Gains and losses of many other chromosomal regions were identified at lower frequencies (Figure 1).

Most regions with recurrent CN changes have previously been found to contain genes that are involved in: (i) colorectal carcinogenesis (i.e., *SMAD4*, *ENO1*, *PIK3CD*, *UBE4B*, *CASZ1*, *CAMTA1*, *PPP1R8*, *TAF12* and *ID3*), (ii) cell growth, survival, proliferation, motility and morphology (*SNHG12* and *LIN28A*), (iii) the metastatic process (*WASF2*, *HTR1D*, *CLIC4*, *LCK*, *PTP4A2*, *ANGPT2*, *ENO1*, *XBP1* and *WNT4*), and (iv) chemoresistance of neoplastic cells (*LIN28A*, *TRPM7*, *NRG1* and *RBBP4*) (Table 2). In turn, the CN regions contained three known microRNAs (MIR34A, MIR367 and MIR302A) that regulate the expression of genes involved in the pathogenesis of colorectal cancer (Table 2).

### 3.3. Chromosomal Alterations and Response to Preoperative RCT

**Figure 2:**
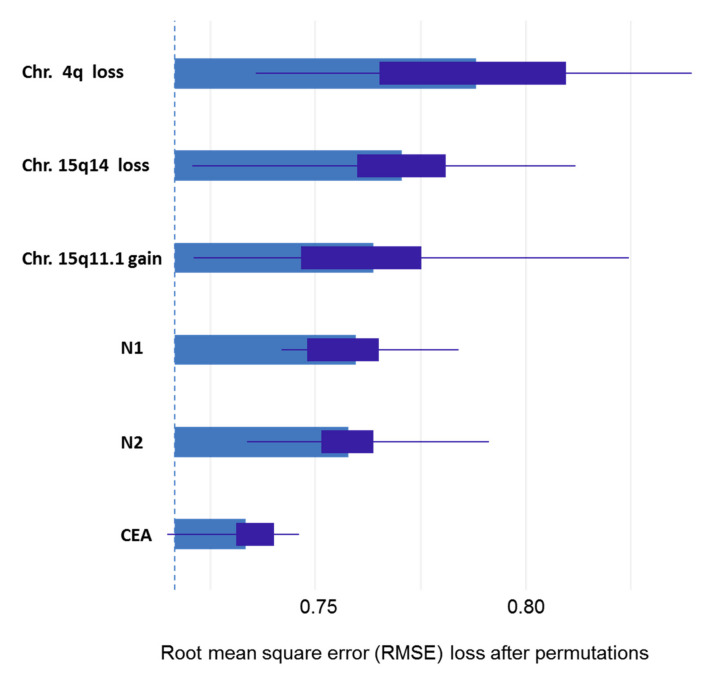
Summary of DALEX method results. Degree of importance of the clinical, biological, and genetic characteristics previously selected by the five algorithms that contributed the most to predicting the Dworak grade of tumor regression after RCT was administered before surgery. The variables are arranged in order of increasing importance. The results show that chromosomal alterations in 4q, 15q14, and 15q11.1 are the most important variables for predicting the response to preoperative RCT in patients with locally advanced rectal cancer.

When studying the association between chromosomal alterations in tumor samples and the degree of response to neoadjuvant therapy, we found significant association between good response (grades 3 and 4 of Dworak) and gain on 8p23.1 (*p* < 0.05). 17q gains were found in both more frequently in responders (G3 and G4); however, this association was not statistically significant (*p* = 0.06) (Table 3). We also performed a second approach to measure the association between these variables and the response. In order to accomplish this task, we used five feature selection algorithms. These algorithms evaluated the degree of importance of the studied variables in the response to preoperative RTC treatment, showing that, both the presence of tumor involvement in ≥4 perirectal lymph nodes (N2) and the 4q loss were the most influential variables in the response to treatment with preoperative RCT, followed by the presence of tumor involvement in 1 to 3 perirectal lymph node (N1), the abnormalities of the 15q11.1, 17q21.31, and 15q14 chromosomal regions, and CEA serum levels (Figure 2 and Table 4). Interestingly, of the five algorithms studied, DALEX and xgboost methods ranked two genetic variables as the most influential in the response to preoperative RCT treatment (Figure 2). We then performed a prediction analysis using five machine learning algorithms with either all the initial variables or the variables selected by their influence on response. Overall, these models were able to predict the 60% of the Dworak grade groups at diagnosis. However, we detected per grade algorithm-wise differences as the models fitted by PLS and Random Forest obtained good results for G2 and G3/G4 grades (100% and 66.7% of hits, respectively), while the wSVM model best predicted the G0/G1 grade (80% of hits) (Table 5).

### 3.4. Analysis of Prognostic Impact and Predictiveness of Clinical-Biologic Features and Chromosomal Alterations on Disease-Free Survival (DFS) an Overall Survival (OS)

**Figure 3:**
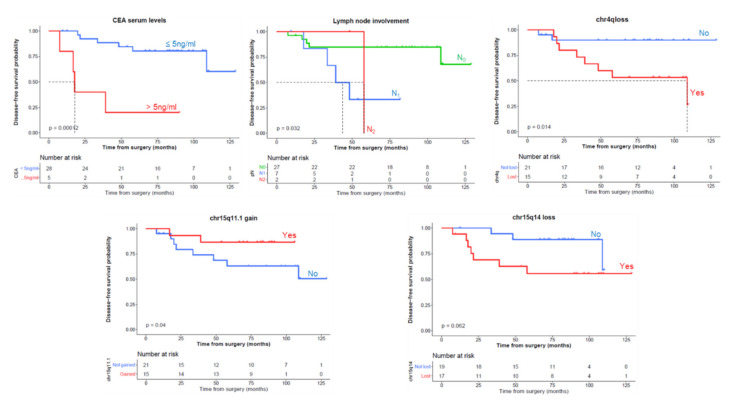
Univariate disease-free survival (DFS) analysis of clinical, biological, and genetic features of locally advanced rectal cancer patients which were selected for multivariate analysis (*p* < 0.05): carcinoembryonic antigen (CEA) serum levels, lymph node involvement, and abnormalities in the 4q, 15q11.1, 15q14, and 17q21.31 chromosomal regions. DFS information was available from 36 cases with a type of resection R0.

**Figure 4:**
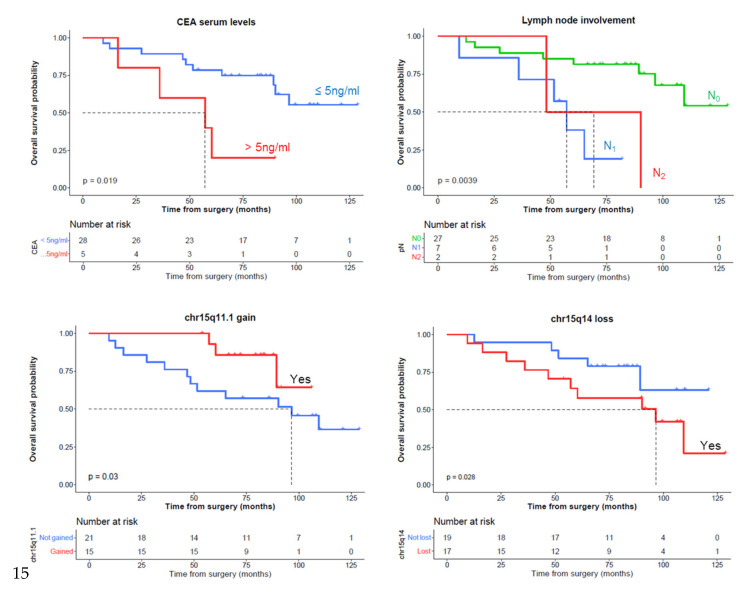
Univariate overall survival (OS) analysis of clinical, biological, and genetic features of locally advanced rectal cancer patients which were selected for multivariate analysis (*p* < 0.05): carcinoembryonic antigen (CEA), lymph node involvement and abnormalities on 15q11.1 and 15q14 chromosomal regions. OS information was available from 36 cases.

**Figure 5:**
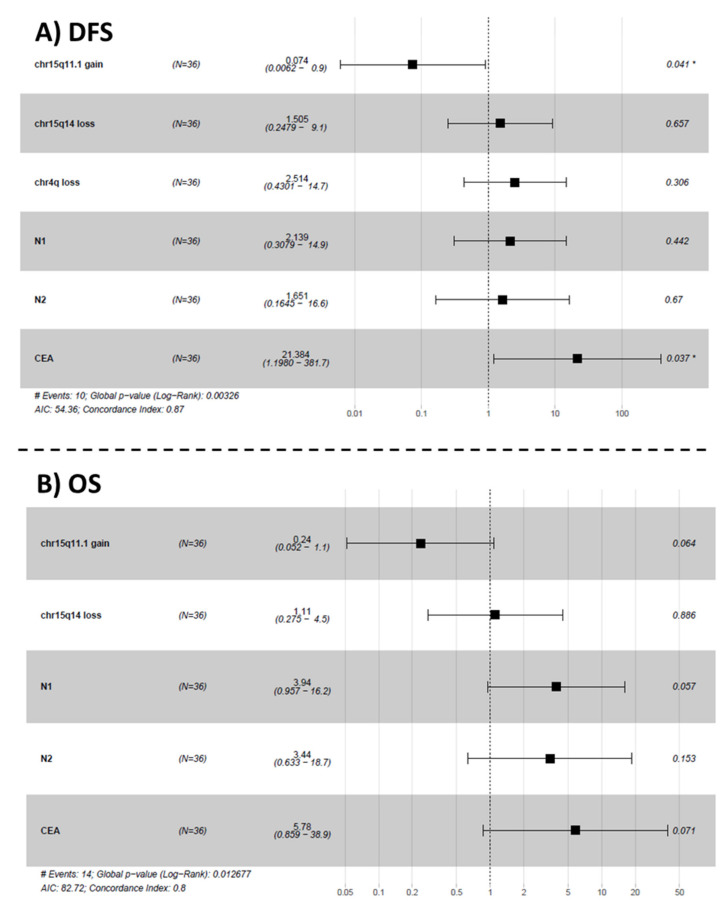
Multivariate analysis of prognostic impact of chromosomal abnormalities showing that gain of the 15q11.1 chromosomal region and CEA serum levels are the only statistically significant independent predictors of disease-free survival (DFS) (Panel **A**). Lymph node involvement (N1) also showed a prognostic impact on overall survival (OS) (Panel **B**) in the multivariate analysis. Survival analysis parameters are indicated with #.

In the first stage, we selected minimal common regions (MCRs) with a higher degree of association with DFS and OS according to a univariate survival analysis (*p*-value < 0.15). Interestingly, from a prognostic viewpoint, the selected MCRs consistent in losses of chromosomes 4q and 15q14 showed a higher incidence of relapses together with a shorter DFS, while the gains at 15q11.1 and 17q21.31 chromosomal regions displayed a lower incidence of relapses and longer DFS (Figure 3). Losses on 15q14 and the absence of gains in 15q11.1 and 17q21.31 were associated with lower OS (Figure 4). As expected, CEA serum levels >5ng/mL and lymph node involvement were significantly associated with a worse DFS and OS (Figure 3 and Figure 4, respectively). All these variables were used to fit a Cox multivariate model that showed that gains on 15q11.1 and CEA levels serum at diagnosis were the only independent variables for DFS. In addition to these two variables, lymph node involvement (N1) also showed a prognostic impact on OS in the multivariate analysis (Figure 5). With the variables analyzed for survival in the multivariate analysis, we developed a deep-learning-based prediction model for predicting survival rates of LARC patients. The studies had a 100% success rate for predicting both DFS and OS at 60 months after diagnosis of the disease (Table 6 and Table 7, respectively).

### 3.5. Correlation between the Chromosomal Changes Detected by the SNP Array and FISH Techniques

To evaluate the consistency of the chromosomal changes identified by the SNP arrays, FISH analysis was performed in parallel for five chromosomal regions from four chromosomes. We found a close correlation overall (mean r^2^ = 0.81 ± 0.04; range: 0.76–0.86) between the two methods, even when the analysis was restricted to the most frequently altered regions (r^2^ ≥ 0.76) (Supplementary Table S3).

## 4. Discussion

The administration of neoadjuvant radiochemotherapy (RCT) followed by surgery has become standard clinical practice for the treatment of locally advanced rectal cancer (LARC) patients [31]. Although this treatment strategy is associated with an overall benefit to patients [32], the degree of response to RCT varies considerably, not only among patients but even between clinically identical tumors. In fact, around 20–30% of cases do not respond to therapy and some of them may even show disease progression [33]. At present, it is not known which tumors are more sensitive or resistant to neoadjuvant RCT, and which factors determine good or poor responses to RCT administered before surgery. In addition to the clinical and biological characteristics, the genetic alterations of tumor cells have been suggested to play a role [29,30,31] due to the great genetic heterogeneity of tumor cells between and within tumors [34], as we have previously seen [12]. In this study, we construct a comprehensive map of the genetic alterations present in LARC through the use of high-resolution SNP arrays, with a median distance between interrogated single-nucleotide polymorphisms (SNPs) of 680 bases; our primary goal was to gain insight into the most frequent genetic alterations that could be associated with response or resistance to neoadjuvant therapy, as well as with survival of the disease.

It is important to highlight that the (copy number) CN changes in our series were assessed by comparing the intensity distribution to a reference set of normal samples from healthy individuals with negative colonoscopies from the Castilla y León (Spain) region, whereas most other studies carried out to date have used individuals from the international HapMap project (https://www.thermofisher.com/es/es/home/life-science/microarray-analysis/microarray-data-analysis/genechip-array-library-files.html, accessed on 23 June 2021) as reference [33,34,35,36]. Interestingly, when we used individuals from the international HapMap project (unpublished data) as reference, our series showed a higher incidence than in healthy controls from Castilla y León of gains of 2p11.2 (92% vs. 13% of cases), 7q36 (56% vs. 15%), 14q32 (97% vs. 13%), and 22q11 (72% vs. 5%) and losses of 15q24 (62% vs. 43%) 8p11 (67% vs. 51%), and 17p11.2 (77% vs. 43%). This was probably a consequence of the variation in the haplotypes present within the different ethnic groups of the HapMap samples [37]. This limitation of HapMap studies should be kept in mind because of their enormous potential for generating false-positive associations.

Previous reports have repeatedly identified a high frequency of gains of chromosomes 7, 8q, 13q, 14q, and 20 and losses of the 1p, 5q, 8p, 14q, 15q, 17p, and 18q chromosomal regions [10,38,39,40] in LARC patients. Consistent with these observations, all rectal cancer tumor samples obtained at diagnosis (before therapy) showed complex karyotypes with at least four altered chromosomes. As previously described, the most common alterations observed included gains of chromosomes 8q, 15q, and 20p and losses of the 1p, 4q, 8p, 15q, and 18q chromosomal regions. Most of these chromosomal abnormalities were present at similar frequencies in all groups of patients defined according to their response to therapy (e.g., Dworak grades). However, important differences were identified between cases showing different degrees of response to neoadjuvant therapy. Del(1p) predominated among the responder patients (Dworak grades 3 and 4), whereas losses of 3q22, 7q34, 7q35, and 12p11.23 were more frequent in the non-responders (Dworak grades 0 and 1). Together, these results suggest that response to RCT neoadjuvant is associated with specific chromosomal alterations. Further studies are necessary to determine the exact molecular mechanisms involved in tumor cell sensitivity and resistance to therapy. Genes involved in the chemoresistance process of neoplasic cells are found in these chromosomal regions, particularly in the 1p region. Fang et al. found that *LIN28A* activation, located at 1p36, contributes to the chemoresistance of liver cancer [40]. Wang et al. also found that the *RBBP4* gene (1p35) is associated with platinum chemoresistance in lung adenocarcinoma [41].

In addition, the predictive analytic statistical methods showed that the alterations on 4q, 15q11.1,15q14, and 17q21.31 were the best combination of genetic variables for predicting the response to treatment with RCT prior to surgery, as occurs in other tumors [42,43,44,45]. In line with our observations, Chen et al. found that loss of chromosomal region 15q was significantly associated with an absence of pathological response in patients with LARC [13]. Of the losses detected on the q arm of chromosome 15, we found a small region of chromosomal gain of 130,698 base pairs in the 15q11.1 band, in the 41% of the studied, that is associated with a good response to neoadjuvant treatment and a favorable prognosis. In this minimal common region (MCR) gained, only the *HERC2P3* gene is located, one of the *HERC2* pseudogenes (an E3 ubiquitin protein ligase). Its role and expression in cancer are still unclear. However, Chen et al. [44] demonstrated that *HERC2P3* plays a critical role in cell growth and migration in human gastric cancer cells, suggesting that *HERC2P3* may serve as a potential biomarker for diagnosis and treatment of gastric cancer [44]. Bonanno et al. [45] reported that *HERC2P3* has a predictive role in advanced non-small-cell lung carcinoma (NSCLC). In the present study, gain of the 15q11.1 chromosomal region was an independent predictor of disease free survival (DFS) and overall survival (OS). In this regard, *HERC2P3* gene status could be considered a new candidate marker to evaluate the tumor response to RCT before surgery and the progression of disease in patients with LARC. Additional prospective studies in larger series of patients would be required to confirm the clinical utility of this new marker. Consistent with previous observations [46,47,48,49,50,51], which show that abnormally high carcinoembryonic antigen (CEA) serum levels are associated with poor prognosis in LARC patients, our study also showed the same association in the multivariate analysis of DFS and OS. Some other features, such as lymph node involvement and losses in the 4q and 15q14 chromosomal regions, were prognostically relevant solely in the univariate analyses. The entire 4q arm has previously been identified as the site of several putative tumor suppressor genes in various tumor types [52,53], including colorectal cancer [50]. Consistent with other studies, and using similar methods, our study discovered a loss of 4q in almost half of the LARC cases examined [10,38,39]. However, CN alterations of chromosomes 4q and 15q14 have been associated with clinical outcome only in studies of colorectal cancer patients; no studies have focused specifically on rectal cancer. Brosens et al. [50] reported significant relapses in patients in stage II with losses at chromosome 4q, suggesting that these genomic alterations could be used to help select patients for adjuvant therapy. Bardi et al. [51] also found a significant association between loss at chromosome 4q and worse DFS in univariate analyses. We confirmed that 4q loss is a relatively frequent genetic event in LARC patients, associated with a poor response to neoadjuvant treatment in LARC and an adverse prognosis, early relapse and short survival. Recently, Kobayashi et al. presented evidence of the possible existence of additional tumor suppressor genes (e.g., *PAICS* gene) located on the 4q chromosomal region in microsatellite stable but chromosomally unstable tumors of CRC patients [52]. Similarly, accumulation of alterations on 15q is strongly associated with adenoma-to-carcinoma progression in CRC patients, independent of the degree of dysplasia [53]. Moreover, Sheffer et al. linked worse prognosis to a simultaneous deletion of 4q, 15q and 18q in colorectal cancer [54]. In all these studies included both colon and rectal cancer patients and it is well known that there are differences between genetic alterations and the location of tumors. Therefore, prognostic indicators in studies of colorectal cancer patients cannot be adequately compared with respect to DFS and SO outcomes in rectal cancer patients.

In addition to the association here described, other authors have studied different biomarkers associated with clinical outcome, such as the expression of *HER2*, *MIR31*, *EGFR*, *VEGF*, and mismatch repair (MMR) genes. El Otmani et al. [55] reported that a high expression of *HER2* by immunohistochemistry (score 3+) on pretreatment biopsy samples can be a predictive factor of distant metastasis and local recurrence (<2 years). Similarly, Caramés et al. [56] proposed quantification of the miR-31 levels as a novel valuable clinical tool to predict both pathological response and outcome in LARC patients. The association of higher *EGFR* and *VEGF* expression with unfavorable outcome in LARC patients has been described by several authors [57,58]. Another biomarker that has been related to survival is MMR deficiency. Huh et al. [59] showed that MSH6 protein expression is an independent predictor for OS in pretreatment biopsy tissue. Despite these advances, local recurrence and distant metastasis remain an issue, with one-third of LARC patients dying within five years of initial treatment [60]. Identification of predictive biomarkers in patients with LARC may help make decisions in the postoperative management strategies to improve patient outcomes [61].

There are currently trials underway to treat patients with locally advanced rectal cancer with a new treatment regimen called total neoadjuvant therapy (TNT). The TNT approach consists of the addition of induction and consolidation, so that all systemic chemotherapy and neoadjuvant RCT are administered prior to surgery. This scheme improves treatment tolerance and compliance and early treatment of micrometastases. In addition, TNT has shown not only better pathological response rates, but also greater rates of complete tumor response, as well as improvements on DFS and OS [62,63]. It would be expected that the findings described in this work would have the same value in predicting the response in patients treated with this new scheme, especially biomarkers detected in the DNA of tumor cells, where mutations associated with resistance or sensitivity to different treatments are harbored. A very clear example is the case of the relationship between RAS mutation status and anti-EGFR therapies (cetuximab or panitumumab) administered before [64] or after [65] the patient’s surgery. However, it would be very interesting to extend the studies to patients included in the trials with TNT.

In the present study, predicting survival using a novel deep-learning-based algorithm featuring the combined assessments of CEA serum levels, lymph node involvement and 4q, 15q11.1,15q14, and 17q21.3 chromosomal region alterations yielded 100% success rates for predicting DFS and OS at 60 months after diagnosis of the disease. To the best of our knowledge, this is the first time that a scoring system based on clinical, biological, and genetic characteristics has been used to identify LARC patients undergoing neoadjuvant treatment who are still at high risk of disease recurrence. If the prognostic value of this new risk stratification model is confirmed in prospective series of LARC patients, it could pave the way for identifying those patients who might genuinely benefit from neoadjuvant RCT in order to be able to perform conservative surgery.

## 5. Conclusions

In summary, we used high-resolution SNP array techniques to describe in detail the genetic alterations associated with clinical outcome and response to RCT administered prior to surgery, as assessed by the Dworak regression system. Further gene expression profiling and functional studies focusing on the genes situated on chromosomes 4q, 15q11.1, 15q14, and 17q21.31, and their potential interactions, are needed to determine the exact molecular mechanisms involved in such associations and to develop ways of reversing them.

## Figures and Tables

**Table 1:** Clinical and biological characteristics of locally advanced rectal cancer patients (*n* = 39) before and after treatment (radiochemotherapy) administered prior to surgery.

Clinical Features	Pre-Treatment	Post-Treatment	*p*
Age (years) *	69 (39–88)	69 (39–88)	NS
Gender Female Male	11 (28%) 28 (72%)	NA NA	NA
Tumor Size (cm) *	4 (1–5)	1.92 (0–4)	0.02
Localization in the rectum Lower Medium Upper	4 (10%) 20 (51%) 15 (39%)	NA NA NA	NA
TNM T0 T1 T2 T3 T4	0 (0%) 0 (0%) 1 (2%) 26 (67%) 12 (31%)	5 (13%) 3 (8%) 14 (36%) 17 (43%) 0 (0%)	<0.0001
N0 N1 N2	8 (20%) 30 (77%) 1 (3%)	27 (69%) 10 (26%) 2 (5%)	<0.0001
M0 M1	39 (100%) 0 (0%)	38 (97%) 1 (3%)	NS
Tumor stage Stage 0 Stage I Stage II Stage III Stage IV	0 (0%) 1 (3%) 6 (15%) 32 (82%) 0 (0%)	4 (10%) 15 (39%) 8 (20%) 11 (28%) 1 (3%)	<0.0001
Dworak regression grade G0 G1 G2 G3 G4	NA NA NA NA NA	3 (8%) 13 (33%) 13 (33%) 5 (13%) 5 (13%)	NA
Type of surgery APR AR	NA NA	13 (33%) 26 (67%)	NA
Type of tumor resection R0 R1 R2	NA NA NA	36 (92%) 1 (3%) 2 (5%)	NA
CEA serum levels ≤5 ng/mL ≥5 ng/mL	23 (59%) 16 (41%)	34 (87%) 5 (13%)	0.005
*KRAS* mutation Wild-type Mutated G12D G12V G13D	26 (67%) 1 (3%) 3 (8%) 4 (10%) 5 (12%)	NA NA NA NA NA	NA
Local recurrence	NA	2 (5%)	NA

Results are expressed as number (percentage) of cases or, where indicated with *, as the median (range). Pre-treatment tumor size and TNM pre-treatment status were determined by image techniques; TNM post-treatment status was determined by histopathology after preoperative radiochemotherapy (RCT). The response was measured on the Dworak regression grading system: grade 0, no regression; grade 1, dominant tumor mass with obvious fibrosis and/or vasculopathy; grade 2, dominantly fibrotic changes with few tumor cells or groups of tumor cells; grade 3, very few tumor cells in fibrotic tissue, with or without mucous substance, and; grade 4, no tumor cells, only fibrotic mass (total regression or response). APR: abdominoperineal resection; AR: anterior resection. R0: distal and circumferential verges without tumor cells; R1: distal or circumferential verges with tumor cells; R2: distal and circumferential verges with tumor cells. CEA: carcinoembryonic antigen. NA: not applicable. NS: not statistically significant (*p* > 0.05).

**Table 2:** Most frequently detected minimal common regions (*q*-value < 0.01 and frequency > 40% of altered cases) of gain and loss in locally advanced rectal cancer tumors genotyped on the Affymetrix 6.0 SNP array platform (*n* = 39).

Minimal Common Altered Regions (bp)	Region Length (bp)	N. of SNPs	Chr. Band	Event	Altered Cases (%)	Gene List
Chr18: 48351659-48920677	569018	2340	18q21.2	Loss	69	*RN7SL695P*, *SRSF10P1*, *RNU1-46P*, *MRO*, *ME2*, *MEX3C*, *ELAC1*, ***SMAD4***
Chr20: 1560988-1585059	24071	18	20p13	Gain	56	*SIRPB1*
Chr1: 7829422-10869532	3040110	14577	1p36.23	Loss	56	*RNU1-7P*, *RN7SL729P*, *RNU6-991P*, *RPL7P11*, *RPL7P7*, *ENO1-IT1*, *ENO1-AS1*, *RNU6-304P*, *HMGN2P17*, *RN7SL451P*, ***MIR34A***, *RNA5SP40*, *C1orf200*, *RN7SKP269*, *MIR5697*, *PGAM1P11*, *RNU6-828P*, *MIR1273D*, *RNU6-37P*, *RN7SL731P*, *RN7SL721P*, *CORT*, *RN7SL614P*, *VAMP3*, *UTS2*, *PARK7*, *ERRFI1*, ***ENO1***, *CA6*, *SLC2A7*, *SLC2A5*, *SPSB1*, *SLC25A33*, *TMEM201*, ***PIK3CD***, *LZIC*, *NMNAT1*, *RBP7*, *PGD*, *APITD1*, *APITD1-CORT*, ***DFFA***, ***PER3***, *TNFRSF9*, *RERE*, *GPR157*, *H6PD*, *CLSTN1*, *CTNNBIP1*, ***UBE4B***, *KIF1B*, *PEX14*, ***CASZ1***, ***CAMTA1***, *SLC45A1*
Chr1: 26284282-31197400	4913118	20676	1p35.3	Loss	54	*RNU6-110P*, *SLC30A2*, *FAM110D*, *ZNF593*, *CD52*, *RN7SL490P*, *HMGN2*, *DPPA2P2*, *MIR1976*, *RN7SL679P*, *RN7SL501P*, *RN7SL165P*, *SFN*, *GPATCH3*, *NR0B2*, *OSTCP2*, *TRNP1*, ***FAM46B***, *CHCHD3P3*, *NPM1P39*, *SNRPEP7*, *RNU6-48P*, *FCN3*, *CD164L2*, *IFI6*, *RNU6-949P*, *CHMP1AP1*, *RNU6-424P*, *RPEP3*, *RNU6-1245P*, *SCARNA1*, *THEMIS2*, *XKR8*, *RN7SL559P*, *SPCS2P4*, *RNU6-176P*, *RNU7-29P*, *ATPIF1*, *RNU6ATAC27P*, *SNORA73B*, *PRDX3P2*, ***SNHG12***, *SNORD99*, *RAB42*, *RNU11*, *TMEM200B*, *PAFAH2*, *EXTL1*, *TRIM63*, *PDIK1L*, *CNKSR1*, *CATSPER4*, *CEP85*, *UBXN11*, *AIM1L*, *ZNF683*, *DHDDS*, ***ARID1A***, *PIGV*, *ZDHHC18*, *GPN2*, *C1orf172*, *SLC9A1*, ***WDTC1***, *SYTL1*, *MAP3K6*, *GPR3*, *FGR*, *FAM76A*, *STX12*, ***PPP1R8***, ***RPA2***, *SMPDL3B*, *PTAFR*, *DNAJC8*, ***SESN2***, *MED18*, *TRNAU1AP*, *GMEB1*, *YTHDF2*, *OPRD1*, *MECR*, *SH3BGRL3*, ***LIN28A***, ***RPS6KA1***, *TMEM222*, ***WASF2***, *AHDC1*, *PHACTR4*, *RCC1*, ***SNHG3***, ***TAF12***, *SRSF4*, *PTPRU*, *MATN1*, *MATN1-AS1*, *NUDC*, *EYA3*, *EPB41*
Chr1: 23401844-25226751	1824907	7976	1p36.11	Loss	54	*RNU6-514P*, *RNU6-135P*, ***HTR1D***, *C1orf213*, ***ID3***, *RN7SL532P*, *PITHD1*, *LYPLA2*, *GALE*, *RN7SL24P*, *MIR378F*, *PNRC2*, *RN7SL857P*, *RNU6-1208P*, ***KDM1A***, *HNRNPR*, *ZNF436*, ***ASAP3***, *MDS2*, *RPL11*, ***TCEB3***, ***HMGCL***, ***FUCA1***, ***SRSF10***, *MYOM3*, *IL22RA1*, ***GRHL3***, *STPG1*, *RCAN3*, *SRRM1*, ***RUNX3***, *LUZP1*, *TCEA3*, ***E2F2***, *CNR2*, *IFNLR1*, *NIPAL3*, *NCMAP*, ***CLIC4***
Chr8: 39235592-39384956	149364	964	8p11.22	Gain	51	*ADAM5*, *ADAM3A*
Chr1: 20830489-20979684	149195	963	1p36.12	Loss	51	*MUL1*, *RPS4XP4*, *FAM43B*, ***CDA***, *DDOST*, ***PINK1***, *PINK1-AS*
Chr1: 31457917-31735879	277962	1756	1p35.2	Loss	51	*SEPW1P*, *NKAIN1*, *SNRNP40*, *PUM1*
Chr15: 34670991-34830240	159249	1137	15q14	Loss	49	*MIR1233-1*, *HNRNPLP2*, *MIR1233-2*, *GOLGA8A*, *GOLGA8B*
Chr15: 50557160-51352248	795088	3936	15q21.2	Loss	49	*MIR4712*, *AHCYP7*, *RNA5SP395*, *RN7SL354P*, *DCAF13P3*, ***HDC***, *GABPB1-AS1*, *USP50*, *SPPL2A*, *GABPB1*, *USP8*, ***TRPM7***, *AP4E1*, ***TNFAIP8L3***
Chr1: 32278463-33614161	1335698	4306	1p35.1	Loss	48	*MIR5585*, *IQCC*, *DCDC2B*, ***EIF3I***, *FAM167B*, *FAM229A*, *GAPDHP20*, *LRRC37A12P*, *RN7SL122P*, *FNDC5*, *TMEM54*, *SPOCD1*, *TMEM39B*, *TXLNA*, *CCDC28B*, *TMEM234*, *MTMR9LP*, ***LCK***, *MARCKSL1*, *TSSK3*, *BSDC1*, *ZBTB8B*, *ZBTB8OS*, ***RBBP4***, *KIAA1522*, *YARS*, ***HPCA***, *AK2*, *TRIM62*, ***PTP4A2***, *KPNA6*, ***HDAC1***, *ZBTB8A*, *SYNC*, *S100PBP*, *RNF19B*, ***KHDRBS1***, *ADC*
Chr8: 2784419-6422612	3638193	44962	8p23.1	Loss	48	*RNA5SP251*, *RN7SL872P*, *PAICSP4*, *RN7SL318P*, *RPL23AP54*, *RN7SKP159*, ***ANGPT2***, ***CSMD1***, ***MCPH1***
Chr8: 32577483-35655135	3077652	13569	8p12	Loss	48	*RNU6-663P*, *MTND1P6*, *MTND2P32*, *RANP9*, *RNU6-528P*, *SNORD13*, *RN7SL621P*, *RN7SL457P*, *VENTXP5*, *LSM12P1*, *TTI2*, *MAK16*, *DUSP26*, *FUT10*, *RNF122*, ***NRG1***, ***UNC5D***
Chr1: 17005967-17253362	247395	1356	1p36.13	Loss	46	*EIF1AXP1*, *FAM231C*, *RNU1-4*, *CROCCP4*, *MIR3675*, *RNU1-2*, *MST1L*, *ESPNP*, *CROCC*
Chr15: 35085898-35540410	454512	2309	15q14	Loss	46	*ACTC1*, ***NANOGP8***, *PRELID1P4*, *ZNF770*, *AQR*, *ANP32AP1*, *DPH6*
Chr4: 113427910-113740790	312880	1219	4q25	Loss	46	*NEUROG2*, *MIR302B*, ***MIR367***, *MIR302D*, ***MIR302A***, *MIR302C*, *WRBP1*, *RPL7AP30*, *LARP7*, *OSTCP4*, *C4orf21*, *ANK2*
Chr4: 165303804-166130292	826488	5907	4q32.3	Loss	46	*RNU6-284P*, *RNU6-668P*, *TRIM60P14*, *FAM218BP*, *NACA3P*, *FAM218A*, *TRIM61*, *TRIM60*, *TMEM192*, *KLHL2*, *MARCH1*
Chr22: 29192671-29455689	263018	1166	22q12.1	Loss	46	*C22orf31*, ***XBP1***, *ZNRF3-IT1*, *ZNRF3-AS1*, ***ZNRF3***
Chr17: 44267864-44276547	8683	56	17q21.31	Loss	44	*KANSL1-AS1*, *KANSL1*
Chr14:1-20456201	20456200	4929	14q11.2	Loss	44	*RNU6-458P*, *OR11H12*, *ARHGAP42P5*, *NF1P4*, *MED15P1*, *RNU6-1239P*, *GRAMD4P3*, ***DUXAP10***, *OR11H13P*, *GRAMD4P4*, *RNU6-1268P*, *MED15P6*, *ARHGAP42P4*, *OR11H2*, *OR4Q3*, *OR4H12P*, *OR4M1*, *OR4N1P*, *OR4K3*, *OR4K2*, *OR4K4P*, *OR4K5*, *OR4K1*, *OR4K16P*, *OR4K15*, *POTEG*, *BMS1P17*, *BMS1P18*, *POTEM*, *OR4N2*, *OR11K2P*, *OR4K6P*
Chr4: 128751602-129198401	446799	1425	4q28.2	Loss	44	*RNU6-583P*, *FOSL1P1*, ***PLK4***, *C4orf29*, *PGRMC2*, *HSPA4L*, *MFSD8*, *LARP1B*
Chr1: 152552808-152586527	33719	100	1q21.3	Loss	41	*LCE3D*, *LCE3C*, *LCE3B*
Chr1: 22455143-22963470	508327	2714	1p36.12	Loss	41	*MIR4418*, *ZBTB40-IT1*, *C1QA*, ***WNT4***, ***EPHA8***, *ZBTB40*
Chr15: 20586675-20717373	130698	443	15q11.1	Gain	41	*HERC2P3*
Chr8: 7290942-7771549	480607	514	8p23.1	Gain	41	*DEFB104B*, *DEFB105B*, *PRR23D1*, *FAM90A6P*, *FAM90A7P*, *FAM90A22P*, *OR7E157P*, *OR7E154P*, *FAM90A14P*, *FAM90A16P*, *FAM90A8P*, *FAM90A17P*, *FAM90A19P*, *FAM90A9P*, *FAM90A10P*, *PRR23D2*, *DEFB107A*, *DEFB105A*, ***DEFB104A***, ***DEFB103A***, ***DEFB4A***, *SPAG11B*, *DEFB107B*, *FAM90A21*, *FAM90A23P*, *FAM90A18P*, *DEFB106A*, *SPAG11*, *HSPD1P2*, *DEFB106B*

Genes that have been commonly associated with colorectal cancer are shown in bold.

**Table 3:** Chromosomal alterations detected at diagnosis in locally advanced rectal cancer tumors (n = 39), which were associated with the grade of tumor regression (Dworak grade) after RCT was administrated prior to surgery.

	Non-Responders (G0 and G1) (*n* = 17)	Partial Responders (G2) (*n* = 9)	Responders (G3 and G4) (*n* = 13)	*q*-Value	Total Cases (*n* = 39)
1p36.12					
Deleted	7 (39%)	4 (44%)	10 (75%)	<0.001	21 (54%)
3q22					
Deleted	1 (7%)	0 (0%)	0 (0%)	<0.001	1 (3%)
7q34					
Deleted	1 (7%)	1 (11%)	0 (0%)	<0.001	2 (5%)
7q35					
Deleted	4 (22%)	2 (22%)	0 (0%)	0.03	6 (15%)
12p11.23					
Deleted	0 (0%)	0 (0%)	1 (8%)	0.04	1 (2.5%)
12p13.31					
Deleted	6 (33%)	0 (0%)	2 (17%)	0.03	8 (21%)
17q21.31					
Deleted	6 (33%)	4 (44%)	8 (58%)	<0.001	18 (46%)
Amplified	7 (39%)	1 (11%)	8 (58%)	<0.001	16 (41%)
20p12					
Deleted	2 (11%)	4 (44%)	5 (42%)	0.001	11 (28%)
22q12.1					
Deleted	5 (28%)	5 (56%)	9 (67%)	0.04	19 (49%)

Results expressed as number of cases and percentage between brackets.

**Table 4:** Analysis of the clinical, biological, and genetic characteristics previously selected by predictive analytics statistical methods, which better contributed to prediction the grade of tumor regression (Dworak grade) after RCT, was administrated prior to surgery.

Variables	Importance Ranking by Method					Median Ranking
	Boruta	Xgboost	Relative Importance	DALEX	VITA	
**N2**	2	7	**1**	3	**1**	2
N1	4	5	4	4	3	4
chr4q loss	3	**1**	3	**1**	2	2
chr15q11.1 gain	5	4	2	2	5	4
chr17q21.31 gain	**1**	2	5	6	7	5
chr15q14 loss	7	3	7	5	6	6
CEA	6	6	6	7	4	6

N1: presence of tumor involvement in 1 to 3 perirectal lymph node; N2: presence of tumor involvement in ≥4 perirectal lymph nodes. CEA: carcinoembryonic antigen determined at diagnosis. The best position of the variable to predict the response to the RCT administrated prior to surgery is shown in bold.

**Table 5:** Dworak response grade prediction analysis. Clinical, biological, and genetic predictors filtering was based on the results of five algorithms which measure the contribution to prediction on the grade of tumor regression (Dworak grade) after RCT was administrated prior to surgery (CEA serum levels, lymph node involvement, and alterations on 4q, 15q11.1, 15q14, and 17q21.31 chromosomal regions).

Algorithm	Parameters	Filtering Method	Nº of Variables	Hit Rate (%)			
				G0/G1	G2	G3/G4	Global
**PLS**	**Number of factors: 3**	**No**	**7**	**40**	**100**	**67**	**60**
PLS	Number of factors: 2	Yes	4	80	0	0	40
**wSVM**	**Kernel: Sigmoid; gamma: 8; cost: 100**	**No**	**7**	**80**	**50**	**33**	**60**
wSVM	Kernel: polynomial; gamma: 0.25; cost: 100	Yes	4	0	0	67	20
SVM	Kernel: Sigmoid; gamma: 0.25; cost: 0.001	No	7	100	0	0	50
SVM	Kernel: polynomial; gamma: 0.25; cost: 0.001	Yes	4	100	0	0	50
KNN	k neighbors: 23	No	7	100	0	0	50
KNN	k neighbors: 23	Yes	4	100	0	0	50
**Random Forest**	**Number of trees: 2**	**No**	**7**	**40**	**100**	**67**	**60**
Random Forest	Number of trees: 2	Yes	4	60	0	33	40

PLS: Partial Least Squares algorithms (SIMFIT software v.6.9.9; www.simfit.org.uk); SVM: Support Vector Machines; KNM: K-Nearest Neighbors; GO/G1: Non-responders; G2: Partial responders; G3/G4: Responders. *we include the 3 or 6 best ranked by the prediction of the analyzed algorithms (N2, chr4q loss, chr15q11.1gain, chr15q14 loss, 17q21.31 gain, N1 and CEA). The best models found to predict the response to the RCT administrated prior to surgery are shown in bold.

**Table 6:** Prediction of DFS at 12, 36, and 60 months based on clinical, biological, and genetic characteristics previously selected in the multivariate analysis for survival (CEA serum levels, lymph node involvement –N1 and N2- and abnormalities on 4q, 15q11.1, 15q14, and 17q21.31 chromosomal regions).

Validation Sample ID	Real Time and Event		Prediction at					
	DFS Censor	Time to DFS (Months)	12 Months		36 Months		60 Months	
			Probability of Absence of the Event	Success in Prediction?	Probability of Absence of the Event	Success in Prediction?	Probability of Absence of the Event	Success in Prediction?
1	1	34	1	YES	0.8	NO	0.5	YES
2	1	18	1	YES	0.0	YES	0.0	YES
3	0	129	1	YES	0.9	YES	0.7	YES
4	1	109	1	YES	0.9	YES	0.9	YES
5	1	8	1	NO	0.0	YES	0.0	YES
6	0	54	1	YES	1.0	YES	1.0	NC
7	0	89	1	YES	1.0	YES	1.0	YES
8	0	86	1	YES	0.9	YES	0.8	YES
9	0	84	1	YES	1.0	YES	1.0	YES
10	0	110	1	YES	1.0	YES	0.9	YES
Sucess rate			90%		90%		100%	
Sensitivity			0%		67%		100%	
Specificity			100%		100%		100%	
Positive predictor value			NC		100%		100%	
Negative predictor value			90%		88%		100%	

NC: not calculable.

**Table 7:** Prediction of overall survival (OS) at 12, 36, and 60 months based on clinical, biological, and genetic characteristics previously selected in the multivariate analysis for survival (CEA serum levels, lymph node involvement, and abnormalities in the 15q11.1, 15q14, and 17q21.31 chromosomal regions).

Validation Simple ID	Real Time and Event		Prediction at					
	OS Censor	Time to OS (Months)	12 Months		36 Months		60 Months	
			Probability of Absence of Event	Success in Prediction	Probability of Absence of the Event	Success in Prediction	Probability of Absence of Event	Success in Prediction
1	1	52	0.9	YES	0.6	YES	0.3	YES
2	1	36	0.0	NO	0.0	YES	0.0	YES
3	0	129	1.0	YES	0.9	YES	0.8	YES
4	0	121	1.0	YES	0.9	YES	0.8	YES
6	1	17	0.0	NO	0.0	YES	0.0	YES
6	0	54	1.0	YES	1.0	YES	1.0	NC
7	1	89	1.0	YES	1.0	YES	1.0	YES
8	0	86	1.0	YES	0.9	YES	0.8	YES
9	0	84	1.0	YES	1.0	YES	1.0	YES
10	0	110	1.0	YES	0.9	YES	0.8	YES
Success rate			NC		100%		100%	
Sensitivity			NC		100%		100%	
Specificity			NC		100%		100%	
Positive predictor value			NC		100%		100%	
Negative predictor value			NC		100%		100%	

NC: not calculable.

## Data Availability

Available from: https://doi.org/10.6084/m9.figshare.14827674.v1, accessed on 23 June 2021.

## Supplementary Materials

The following are available online at https://www.mdpi.com/article/10.3390/cancers13133166/s1, Table S1: Detailed clinical and biological characteristics of each individual locally advanced rectal cancer patient analyzed (*n* = 39)., Table S2: A panel of 5 locus-specific FISH probes directed against 5 different chromosomal regions were used to validate the results obtained with the SNP arrays, Table S3: Locally advanced rectal cancer patients (*n* = 39): correlation between the numerical changes detected by each individual FISH probe used and the copy number changes identified for the corresponding single nucleotide polymorphisms (SNPs) through SNP array studies.
