# Supplementary material for: High-Risk Clinicopathological and Genetic Features and Outcomes in Patients Receiving Neoadjuvant Radiochemotherapy for Locally Advanced Rectal Cancer
Source: Cancers (Basel). 2021 Jun 24;13(13):3166. doi: 10.3390/cancers13133166 (PMC8269103; doi:10.3390/cancers13133166)
Supplement: Supplementary file 1 [file cancers-13-03166-s001.zip › cancers-1231102-supplementary.pdf]

Supplementary Table S1. Detailed clinical and biological characteristics of each individual locally advanced rectal cancer patient analyzed (N=39).

**Cases ID**	**Cases ID**	**Gender**	**Age (years)**	**Tumor size* (cm)**	**Tumor localization**	**Type of surgery**	***u*TNM**	***yp*TNM**	**CEA serum levels (ng/ml)**		***KRAS* mutational status**	**Dworak grade ***
									**Pre-treatment**	**Post-treatment**		
**1**	**1**	M	57	3	MR	APR	T3N1M0	T3N0M0	3.17	3.26	WT	G2
**2**	**2**	M	74	3	UR	AR	T4N1M0	T3N0M0	2.28	2.16	WT	G2
**3**	**3**	M	69	1.5	UR	AR	T3N1M0	T3N0M0	1.8	1.97	G12V	G3
**4**	**4**	M	59	3	UR	AR	T3N1M0	T2N1M0	5.61	1.18	WT	G3
**5**	**5**	F	72	0	MR	AR	T3N1M0	T0N1M0	4.32	1.32	G12V	G4
**6**	**6**	M	75	3	LR	APR	T4N1M0	T3N0M0	8.62	345	G13D	G2
**7**	**7**	F	57	1	UR	AR	T3N1M0	T2N0M0	12.6	1.03	WT	G2
**8**	**8**	M	80	1.5	LR	APR	T3N0M0	T3N1M0	0.58	0.85	NT	G1
**9**	**9**	M	79	3	MR	APR	T3N1M0	T2N0M0	1.52	2.31	WT	G3
**10**	**10**	M	79	3.5	LR	APR	T3N1M0	T1N0M0	2.11	2	G12V	G1
**11**	**11**	M	62	4.7	MR	AR	T3N0M0	T3N1M0	4.56	33.2	WT	G0
**12**	**12**	M	74	3	MR	APR	T3N1M0	T3N2M0	1.13	0.82	WT	G2
**13**	**13**	M	76	1	UR	AR	T3N1M0	T2N0M0	90	1.93	WT	G1
**14**	**14**	M	81	2.5	UR	AR	T3N1M0	T3N1M0	26.8	26.8	G13D	G2
**15**	**15**	M	85	4	LR	APR	T4N1M0	T2N1M0	1.44	1.63	WT	G0
**16**	**16**	F	45	2.5	UR	AR	T4N1M0	T3N1M0	1.95	2.11	WT	G2
**17**	**17**	F	88	2	MR	AR	T4N0M0	T3N0M0	0.9	0.87	NT	G3
**18**	**18**	M	62	0.4	UR	AR	T3N0M0	T2N0M0	1.49	1.52	WT	G1
**19**	**19**	M	65	3.5	MR	AR	T3N0M0	T3N0M0	16	10.1	WT	G2
**20**	**20**	M	57	2.8	MR	APR	T3N1M0	T2N0M0	1.45	1.76	WT	G1
**21**	**21**	M	81	4	MR	AR	T4N2M0	T3N1M1	126	5.05	WT	G1
**22**	**22**	M	85	3	UR	AR	T4N1M0	T3N0M0	46.6	2.42	WT	G1
**23**	**23**	F	72	2	UR	AR	T4N1M0	T3N1M0	3.06	0.8	WT	G2
**24**	**24**	M	74	4.5	MR	APR	T4N1M0	T1N0M0	10.75	1.01	G13D	G1
**25**	**25**	M	60	0	MR	AR	T3N1M0	T2N0M0	8.13	2.22	NT	G3
**26**	**26**	M	78	2.8	MR	APR	T3N1M0	T2N0M0	196	2.17	WT	G2
**27**	**27**	M	81	0	MR	AR	T3N1M0	T1N0M0	6.45	3.24	WT	G1
**28**	**28**	F	60	2.4	MR	AR	T3N1M0	T3N1M0	2.6	2.7	G12V	G0
**29**	**29**	F	53	2.5	MR	AR	T3N1M0	T2N0M0	3.89	2.61	G12V	G2
**30**	**30**	F	75	2	UR	AR	T3N1M0	T2N0M0	3.22	0.58	WT	G1
**31**	**31**	M	66	4	MR	AR	T3N1M0	T3N2M0	6.25	1.47	WT	G2
**32**	**32**	M	53	3	UR	AR	T3N1M0	T3N0M0	1.31	1.33	WT	G1
**33**	**33**	M	39	2	UR	AR	T2N0M0	T2N0M0	0.95	1.03	G12V	G1
**34**	**34**	M	60	2	MR	APR	T4N1M0	T2N0M0	2.16	2.22	WT	G1
**35**	**35**	M	66	0	UR	AR	T3N1M0	T0N0M0	7.22	3.71	WT	G4
**36**	**36**	F	82	0	UR	AR	T3N0M0	T0N0M0	1.7	1.9	WT	G4
**37**	**37**	F	65	0	MR	APR	T4N1M0	T0N0M0	36	4.7	G12V	G4
**38**	**38**	M	67	0	MR	APR	T4N1M0	T0N0M0	6.15	4.45	WT	G4
**39**	**39**	F	80	1.3	MR	AR	T3N1M0	T2N0M0	2.44	1.22	G13D	G2

M: male; F: female; LR: lower rectum (0-4 cm from the anal verge); MR: medium rectum (5-8 cm the anal verge); UR: upper rectum (9-12 cm the anal verge); APR: abdominaeperineal resection; AR: anterior resection; *u*TNM: pre-treatment T stage and lymph node and metastatic status determined by imaging techniques; *yp*TNM: TNM stage determined by histopathology after radiochemotherapy; CEA: carcinoembryonic antigen; WT: wild-type; NT: not tested. * Tumor size at surgery, after radiochemotherapy

Supplementary Table S2. A panel of five locus-specific FISH probes directed against five chromosomal regions was used to validate the results obtained with the SNP arrays.

FISH probe chromosome localization	FISH probe length (kb)	Target gene	Number of SNPs inside the region identified by FISH probe
1p36	110	*P58*	193
8p22	170	*LPL*	46
8q24	600	*CMYC*	203
17p13	145	*TP53*	17
18q21	750	*BCL2*	212

Supplementary Table S3. Locally advanced rectal cancer patients (n = 39): correlation between the numerical changes detected by each individual FISH probe used and the copy number changes identified for the corresponding single nucleotide polymorphisms (SNPs) by SNP array studies.

Chromosomal region identified by FISH probe	R^2/p
1p36	0.81/<0.001
8p22	0.86/<0.001
8q24	0.80/<0.001
17p13	0.81/<0.001
18q21	0.76/<0.001
